# Guidelines for breast cancer management in Bosnia and Herzegovina

**DOI:** 10.17305/bjbms.2022.7504

**Published:** 2023-01-06

**Authors:** Lejla Hadžikadić-Gušić, Timur Cerić, Inga Marijanović, Ermina Iljazović, Dijana Koprić, Anela Zorlak, Mahira Tanović, Alma Mekić-Abazović, Ibrahim Šišić, Una Delić, Jasminka Mustedanagić-Mujanović, Alija Aginčić, Edin Bećiragić, Frederick L Greene

**Affiliations:** 1 Department of Surgical Oncology, Levine Cancer Institute, Atrium Health, Charlotte, NC, USA; 2 Department of Medical Oncology, University Clinical Center, Sarajevo, Bosnia and Herzegovina; 3 Clinic of Oncology, University Clinical Hospital, Mostar, Bosnia and Herzegovina; 4 Department of Pathology, University Clinical Center, Tuzla, Bosnia and Herzegovina; 5 Department of Medical Oncology and Radiation Oncology, University Clinical Center, Tuzla, Bosnia and Herzegovina; 6 Genetics Counseling, Genetika, Sarajevo, Bosnia and Herzegovina; 7 Plastic and Reconstructive Surgery, Advanced Plastic Surgery of North Shore, NY, USA; 8 Department of Medical Oncology, Kanton Hospital Zenica, Zenica, Bosnia and Herzegovina; 9 Department of Radiology, University Clinical Center, Sarajevo, Bosnia and Herzegovina; 10 Plastic and Reconstructive Surgery, Center for Aesthetic Surgery, Nasa Mala Klinika (Our Little Clinic), Sarajevo, Bosnia and Heregovina; 11 Department of Surgery, Dr. Abdulah Nakas General Hospital, Sarajevo, Bosnia and Herzegovina; 12 Cancer Data Services, Levine Cancer Institute, Charlotte, NC, USA

**Keywords:** Breast cancer, Bosnia and Herzegovina (BiH), treatment, guidelines, consortium, breast, multidisciplinary

## Abstract

Breast cancer is the most common cancer among women. In Bosnia and Herzegovina (BiH), accurate data on the status of breast cancer are lacking due to the absence of a central registry. Multiple international guidelines imply that institutions that monitor breast cancer patients should have optimal therapeutic options for treatment. In addition, there have been several international consensus guidelines written on the management of breast cancer. Application of consensus guidelines has previously been demonstrated to have a positive influence on breast cancer care. The importance of specialty breast centers has previously been reported. As part of the 2021 Bosnian-Herzegovinian American Academy of Arts and Sciences (BHAAAS) conference in Mostar, a round table of multidisciplinary specialists from BiH and the diaspora was held. All were either members of BHAAAS or regularly participate in collaborative projects. The focus of the consortium was to write the first multidisciplinary guidelines for the general management of breast cancer in BiH. Guidelines were developed for each area of breast cancer treatment and management. These guidelines will serve as a resource for practitioners managing breast cancer in the BiH region. This might also be of benefit to the ministry of health and any future investors interested in developing breast cancer care policies in this region of the world.

## Introduction

Breast cancer is the most common cancer among women. In 2020, the World Health Organization (WHO) announced that 2.3 million women worldwide suffer from breast cancer, with 685,000 deaths worldwide. According to their estimates, by the end of 2020, there will be 7.8 million women who have been diagnosed with breast cancer in the past five years [1]. In Bosnia and Herzegovina (BiH), accurate data on breast cancer are not known. Without a central registry, it is difficult to obtain accurate data on the status of breast cancer in BiH [2]. The management of breast cancer is unique and patient centered. It is divided into three disciplines: surgical oncology, medical oncology, and radiation oncology. Surgical treatment is composed of tumorectomies/lumpectomies and mastectomies of the breast, sentinel axillary biopsies, and axillary dissections. Medical oncology therapy is comprised of chemotherapy, immunotherapy, and anti-hormone therapy. Radiation is typically one of the last treatments, but it can be used at other times as well. The guidelines of the National Comprehensive Cancer Network (NCCN) for the treatment of breast cancer imply that institutions that monitor breast cancer patients should have optimal therapeutic options [3]. There are no optimal therapeutic options in BiH due to financial and organizational reasons.

**Table 1:** Recommendation grading system

**Grade of Recommendation/Description**	**Benefit vs. Risk and Burdens**	**Methodological Quality of Supporting Evidence**	**Implications**
IA/Strong recommendation, high quality evidence	Benefits clearly outweigh risk and burdens, or vice versa	RCTs without important limitations or overwhelming evidence from observational studies	Strong recommendations, can apply to most patients in most circumstances without reservation
1B/Strong recommendation, moderate quality evidence	Benefits clearly outweigh risk and burdens, or vice versa	RCTs with important limitations (inconsistent results, methodological flaws, indirect, or imprecise) or exceptionally strong evidence from observational studies	Strong recommendation, can apply to most patients in most circumstances without reservation
1C/Strong recommendation, low quality evidence	Benefits clearly outweigh risk and burdens, or vice versa	Observational studies or case series	Strong recommendation, but may change when higher quality evidence becomes available
2A/Weak recommendation, high quality evidence	Benefits closely balanced with risks and burden	RCTs without important limitations or overwhelming evidence from observational studies	Weak recommendation, best action may differ depending on circumstances or patients’ or societal values
2B/Weak recommendation, moderate quality evidence	Benefits closely balanced with risks and burden	RCTs with important limitations (inconsistent results, methodological flaws, indirect, or imprecise) or exceptionally strong evidence from observational studies)	Weak recommendation, best action may differ depending on circumstances or patients’ or societal values
2C/Weak recommendation, low quality evidence	Benefits closely balanced with risks and burden	Observational studies or case series	Very weak recommendation, other alternatives may be equally reasonable

RCTs: Randomized clinical trials.

**Table 2:** BHAAAS guideline overview on the management of breast cancer in BiH

**Recommendations**	**Level of Evidence**
Imaging for Breast Cancer	
1. Annual screening mammogram for women of average risk over 40.	IA / Expert opinion
2. MRI is not recommended for routine use for all breast cancer patents. Consider use of MRI in consultation with a radiologist on a per patient basis with consideration of breast density, family history, and genetic predisposition.	IB / Expert opinion
3. Core needle biopsy should be performed where technology and materials are available.	Expert opinion
4. Clips should be placed in the breast and/or axilla where technology and materials are available and where localization techniques are utilized for breast conserving therapy. Clips should not be placed in the axilla if there is no intention of localization for a targeted sentinel lymph node biopsy.	Expert opinion
5. Tumor localization should be performed where technology and materials are available for optimal marking of tumors for breast conserving therapy.	Expert opinion
Imaging for High-Risk Surveillance	
6. Risk modeling should be used for calculation of risk status for women to identify those at a higher risk of developing breast cancer.	2C / Expert opinion
7. Increased surveillance with MRI and/or ultrasound should be considered for women of elevated lifetime risk of developing breast cancer but without a genetic predisposition.	2C / Expert opinion
8. Increased surveillance with MRI and/or ultrasound should be considered for women with a genetic predisposition for developing breast cancer.	1C / Expert opinion
Pathology	
9. Hormone receptor testing should be performed in all cases of breast cancer.	1A / Expert opinion
10. Her2 by IHC or by FISH/ISH testing should be performed in cases of invasive breast cancer.	1A / Expert opinion
11. Residual cancer burden class should be reported after neoadjuvant chemotherapy to help aid in adjuvant decision making.	Expert opinion
Genetics	
12. Genetic testing should be offered where able and where counseling is available in women of higher risk of breast cancer based on a family history or age at diagnosis.	Expert opinion
13. Use of panels should be used when appropriate (consideration of BRCA 1, 2, ATM, CDH1, CHEK2, NBN, NF1, PALB2, STK11).	Expert opinion
14. Adjuvant and neoadjuvant treatment should be tailored to findings of genetic mutations in breast cancer.	IIA / Expert opinion
15. Consideration of prophylactic surgery should be made in women found to have genetic mutations.	Expert opinion
Surgical Oncology-Breast	
16. All specimens removed from the breast should be clearly marked for pathology.	Expert opinion
17. Consider breast conserving therapy in unifocal or multifocal tumors where localization techniques are available in conjunction with the radiologist.	Expert opinion
18. Mastectomy should not be the only treatment offered for breast cancer where localization services and technology are available as well as adjuvant radiation therapy.	IA / Expert opinion
19. Contralateral prophylactic mastectomy should not be performed in all patients with breast cancer undergoing a unilateral mastectomy for breast cancer management.	IA / Expert opinion
20. Immediate reconstruction should be performed where able and oncologically safe for women undergoing mastectomy.	IA / Expert opinion
Surgical Oncology-Axilla (cN0)	
21. A sentinel lymph node biopsy should be performed in clinically node negative patients who are having upfront surgical treatment in centers where dyes for mapping are available for injection and for identification.	IA / Expert opinion
22. Removal of at least three sentinel lymph nodes is recommended when able.	IA / Expert Opinion
23. Intraoperative frozen section is not recommended at the time of upfront surgery for clinically node negative disease (cN0).	IA / Expert opinion
24. The need for further axillary surgery in patients with cN0 disease prior to surgery should be discussed in a multi-disciplinary tumor board after the final pathology is available.	IA / Expert opinion
25. An axillary dissection should be performed in inflammatory breast cancer.	2B / Expert opinion
Surgical Oncology-Axilla (After neoadjuvant therapy)	
26. A sentinel lymph node biopsy should be performed where technically feasible with dyes for mapping available and technology for identification available in the setting of cN1/2 disease after neoadjuvant chemotherapy where there has been a clinical and radiographic response after chemotherapy.	IA / Expert opinion
27. Removal of at least three sentinel lymph nodes after neoadjuvant chemotherapy is recommended when able.	IA / Expert opinion
28. An axillary dissection should be performed for significant axillary burden of disease after neoadjuvant chemotherapy and for locally advanced breast cancer without a significant response to chemotherapy.	Expert opinion
Registry	
29. Use of a central database is strongly encouraged for data tracking and ability to track treatment modalities and recurrence and survival.	Expert opinion
Plastic and Reconstructive Surgery	
30. Consideration of reconstruction should be offered to all women undergoing a mastectomy when oncologically safe.	Expert opinion
31. Omission of immediate reconstruction is recommended in inflammatory breast cancer or advanced breast cancer.	Expert opinion
32. A nipple sparing mastectomy is appropriate to consider when a tumor is greater than 1 cm from the nipple and areolar complex and when the surgeon has adequate training in this operation to minimize postoperative complications such as ischemia.	Expert opinion
Medical Oncology-Neoadjuvant therapy for Early Stage Breast Cancer (I/II)
33. Discussion of patient care in a multidisciplinary tumor board is strongly recommended prior to the start of neoadjuvant chemotherapy.	Expert opinion
34. Consideration should be made for neoadjuvant chemotherapy for all palpable cT2 or larger tumors that are triple negative (TNBC) or Her2 positive.	IB / Expert opinion
35. Consideration of neoadjuvant chemotherapy should be made for cN1 disease of ER/PR positive disease for downstaging of the axilla.	IB / Expert opinion
36. The addition of platinum agents to neo-adjuvant regimens to increase pCR rates for TNBC or BRCA associated tumors should be considered where available.	IIA / Expert opinion
37. Staging scans should be considered prior to the start of neoadjuvant chemotherapy.	Expert opinion
38. It is appropriate to consider TDM1 in the adjuvant setting for women with Her2 positive disease who do not achieve a pathologic complete response.	IIB / Expert opinion
39. It is appropriate to consider Capecitabine in the adjuvant setting for women with TNBC who do not achieve a pathologic complete response.	IIB / Expert opinion
40. Clinical trials should be considered where appropriate for patients and where available.	Expert opinion
Medical Oncology-Adjuvant Considerations for Early Stage Breast Cancer (Stage I/II)	
41. Surgical pathology reports and gene expression profiling (where commercially available) can be considered for decisions on whether to administer adjuvant chemotherapy for hormone positive tumors.	Expert opinion
42. Presentation at a multidisciplinary tumor board should be strongly recommended prior to adjuvant chemotherapy recommendations.	Expert opinion
43. Tamoxifen is strongly recommended for premenopausal women with hormone positive disease or an aromatase inhibitor with ovarian suppression.	IA / Expert opinion
44. All patients with hormone positive disease should receive endocrine therapy as a mainstay of treatment for at least 5 years.	IA / Expert opinion
Metastatic Breast Cancer	
45. The mainstay of treating metastatic breast cancer is the joint decision making of the treating physicians and the patient with consideration of goals of life, maintaining quality of life, general medical state of the patient and burden of disease. The patient and family should be involved in the decision making.	Expert opinion
46. There is no clear role for surgical management of metastatic breast cancer.	2B
47. Intravenous Her2 targeted therapies, either alone or dual therapies with pertuzumab should be considered if the patient has no significant comorbidities.	Expert opinion
48. The mainstay for treatment of metastatic hormone positive disease is hormone therapy. Chemotherapy can be considered if appropriate depending on disease burden and goals of life.	Expert opinion
49. The mainstay of treatment for metastatic triple negative breast cancer (TNBC) is chemotherapy. Bisphosphonates should be considered for bony metastatic disease. The use of PDL1 expression should be considered to guide therapy.	Expert opinion
Radiation Oncology	
50. All women who undergo breast conserving surgery should meet with a radiation oncologist to consider adjuvant radiation therapy.	IA / Expert opinion
51. Hypofractionation is recommended where possible and feasible.	IB / Expert opinion
52. Consider 3D conformal therapy and IMRT where appropriate.	IB / Expert opinion
53. Consider postmastectomy radiation therapy for node positive disease, close or positive margins, high risk disease, or medial tumors.	IB / Expert opinion
54. Consider omission of radiation therapy for women over age 79 with small, ER positive, clinically node negative tumors (cT1N0).	IB / Expert opinion
55. Intraoperative radiation therapy does not have significant long-range studies to support widespread use. More studies are needed.	Expert opinion
Young Women with Breast Cancer	
56. Diagnostic imaging in young women should follow algorithms similar to older women. Consideration should be made for supplemental imaging such as ultrasound or MRI based on breast density or genetic predisposition and discussion with the radiologist.	IIC
57. Routine screening mammogram or any imaging for early detection of breast cancer should not have a role for women under 40 of average risk.	IA / Expert opinion
58. Consideration should be made for a screening breast MRI in young women with a strong family history or found to be at high risk for developing breast cancer or with a genetic predisposition or with a personal history of ionizing radiation to the chest.	IA / Expert opinion
59. The care of young women with breast cancer should be discussed in a multi-disciplinary setting.	Expert opinion
60. Genetic testing should be offered to all young women with breast cancer where available and where a genetic counselor is available for support and counseling.	Expert opinion
61. Young age alone should not be a reason for more aggressive treatment of any modality.	Expert opinion
Breast Cancer in Pregnancy	
62. Consideration should be made for surgical treatment where there is no clear role for neoadjuvant chemotherapy. The preferred timing for surgery is in the 2^nd^ trimester to allow for completion of organogenesis.	Expert opinion
63. The use of Tc99 alone for the purposes of a sentinel lymph node biopsy should be considered when performing this procedure on a pregnant woman if the materials are available. An axillary dissection is not routinely indicated if it is possible to perform a sentinel lymph node biopsy.	Expert opinion
64. Chemotherapy can safely be administered with the help of maternal fetal medicine and should be initiated after the 1^st^ trimester and should be completed by the 35^th^ week of pregnancy.	Expert opinion
65. Tamoxifen should be avoided in pregnancy.	Expert opinion

As part of the 2021 Bosnian-Herzegovinian American Academy of Arts and Sciences (BHAAAS) conference in Mostar, a round table of specialists from BiH and the diaspora was held. All were either members of BHAAAS or regularly participate in collaborative projects. This multidisciplinary consortium consisted of physicians from various disciplines, namely, surgical oncology, medical oncology, radiation oncology, plastic and reconstructive surgery, pathology, radiology, and genetics. The focus of the consortium was to write the first multidisciplinary guidelines for the general management of breast cancer in BiH. Application of consensus guidelines has previously been demonstrated to have a positive influence on breast cancer care [4, 5]. The importance of specialty breast centers has previously been reported [6].

Herein, we present the guidelines for the management of breast cancer by physicians located within the BiH geographical region and those living in the diaspora, as members from BHAAAS or collaborators. Guidelines are presented by cancer management topic.

## Materials and methods

As part of the BHAAAS annual conference, Dr. Hadžikadić-Gušić hosts a breast cancer symposium. In 2021, in part due to the COVID 19 pandemic, this was held virtually. In lieu of a traditional educational symposium, an open round table was held with invited guests from the spectrum of multidisciplinary specialists in the care of breast cancer who were either members of BHAAAS or have participated in several collaborative projects. In addition, this was open to the public and well attended. The guidelines presented, herein, were established at this round table by this consortium of specialists from the BiH region and diaspora. Guidelines were evidence-based and the most recent literature was reviewed per specialty. The grading system used is reported in Table 1 [7]. Statements without use of a grading system were considered standard clinical practice by our panel of experts (Table 2). Consideration was given to regional access to care and therapeutic options when recommendations were made. All authors have commented and approved these guidelines. Dr. Greene was invited as the senior author given his experience in global cancer care, particularly breast cancer, his involvement in the American College of Surgeons, and work both nationally in the United States and abroad.

## GGuidelines by discipline

### Radiology—screening mammography

We highly recommend annual screening mammography for women of average risk under 55 years old. Average risk is defined as women who do not have a personal history of breast cancer, or a strong family history of breast or ovarian cancer. Women 55 years and older of average risk are recommended to have at least a mammogram every two years, but they can be offered an annual mammogram [8–10].

We also strongly recommend screening mammography for all women over 40. In addition, an earlier mammogram may be considered for women who have a family member with breast cancer as such screening should be started 10 years earlier [8–10]. Where possible, consider adding tomosynthesis. This is presently not performed routinely in all regions of BiH.

### Radiology—diagnostic imaging

Once an abnormality is noted on screening mammogram, additional imaging should be considered. This may consist of a diagnostic mammogram with additional mammographic views, tomosynthesis, ultrasound, or magnetic resonance imaging (MRI) technology. We recommend an ultrasound if the mammogram is abnormal or if the woman has a palpable breast tumor that is not visible by mammogram.

We strongly recommend core needle biopsy (CNB), where possible, of both breast and axillary abnormalities. We recommend minimizing excisional or incisional biopsies, where a CNB can be obtained. We strongly recommend placement of titanium clips in the breast and axilla on CNB [11, 12]. At present, this is not routinely performed due to a lack of availability of clips. We strongly recommend the time frame for pathology from CNB to be less than 10 days.

For the aid of surgical therapeutic intervention, we strongly recommend tumor localization before the operation. This requires that a radiologist places a needle guidewire into the tumor before the operation, to guide the surgeon during the lumpectomy or excision. This is currently not routinely performed in all regional locations. In some regions, it is available on request. We recommend this to be an area of focused resource support as it would allow for more breast conservation therapy.

Like the above discussion, we recommend placement of a needle/guidewire into a previously biopsy-proven axillary lymph node that was marked with a clip before the start of chemotherapy and/or for axillary lymph node localization before a sentinel lymph node biopsy (SLNB) [11, 12].

We do not recommend routine MRI for all patients with breast cancer [13–47]. We do recommend consideration of an MRI of the breast in consultation with a specialist in radiological diagnostics in the following cases: need to evaluate extent of disease due to breast density, need to assess enlargement, size, presence of multifocal/multicentric tumor, mammographic occult diseases, occult primary tumors in the case of Paget’s disease of the breast, certain mammographically occult invasive lobular cancers, and need to evaluate the response to neoadjuvant therapy to consider the possibility of cost-effective surgical treatment [13–41, 43–50].

We do recommend MRI in the setting of breast cancer, particularly in the setting to evaluate response to neoadjuvant chemotherapy (NAC) as it has been shown to be the most accurate modality for comparison of residual tumor size compared to pathologic tumor size among other modalities, with a 90% accuracy [48].

It is strongly recommended that all diagnostics be completed within one month after the diagnosis of breast cancer. It is strongly recommended that all patients be presented to a local multidisciplinary committee after diagnosis and before treatment.

### High-risk surveillance

We strongly recommend the Tyrer-Cuzick model for lifelong risk calculation for women [51]. Consider the use of MRI and mammography alternately every six months for women at high risk for breast cancer. High risk is defined as a >20% lifelong risk by risk modeling. Consider the date of birth, breast density, and family history in the calculation.

We strongly recommend the use of MRI for women who have been found to have a pathogenic mutation or clinically actionable variant that elevates the lifetime risk of developing a breast cancer [42, 52, 53].

We also strongly recommend referral of patients to a multidisciplinary commission in the central/city hospital after diagnosis and before treatment in the regional hospital. We suggest a virtual option for the regional council/commission to present patients before treatment in the central/city hospital for the geographic region.

### Pathology

We strongly recommend performing estrogen receptor (ER) and progesterone receptor (PR) immunohistochemistry (IHC) on all malignant breast tumors. We strongly recommend performing Her2 IHC or ISH, whether this is with fluorescence *in situ* hybridization (FISH) or dual color silver *in situ* hybridization (DC-SISH), when necessary, according to the new ASCO-CAP guidelines, on all malignant breast tumors [54–58]. We strongly recommend Ki67 IHC or grade reporting on all malignant breast tumors. We strongly recommend that pathology is available within 10 days after CNB or operation.

We strongly recommend reporting residual cancer burden (RCB) class after NAC.

Consider that the pathologist is available for intraoperative frozen section when appropriate and when it will change the outcome of the operation performed.

We strongly recommend using the latest AJCC standards and WHO classification when reporting stage.

### Genetics

We strongly recommend genetic testing BEFORE treatment for breast cancer in patients identified as having a higher risk, which defined by a positive family history, date of birth, and tumor histology. When possible, we strongly recommend genetic counseling before and after testing for recommendation of panel testing and discussion of results [7, 59, 60]. We recognize that not all centers have genetic counseling available. If possible, reaching out to a nearby genetic counseling center should be attempted.

It is strongly recommended to have *BRCA1/BRCA2* testing for women identified as having a higher risk by the Tyrer–Cuzick risk calculation model. Consider additional genetic panels where appropriate (*ATM, CDH1, CHEK2, NBN, NF1, PALB2, and STK11*) [7, 61, 62].

When performing genetic counseling, it is recommended to take a detailed family history, examining the father’s and mother’s family line, the types of malignancies present in both lines, approximate age at diagnosis, if death occurred and approximately at what age, what treatment was given, and genetic testing (if performed), in addition to external factors that could potentially be identified as triggers [7, 10, 59, 63].

We define high-risk individuals that might benefit from genetic testing to be:

- persons who have a personal or family history of breast or ovarian cancer under the age of 40
- persons who have a personal or family history of breast or ovarian cancer, multiple cancers at a younger age, rare cancers at any age, or cancers associated with the *BRCA1/BRCA2* mutation in one family member.

Genetic testing is recommended in asymptomatic patients with a family history. If the patient is found to carry a higher risk of developing breast cancer, options for risk reducing surgery might be available or a strategy for enhanced surveillance may be warranted [64, 65].

In the setting of breast cancer and a positive *BRCA1/BRCA2* test, we strongly recommend tailoring neoadjuvant or adjuvant chemotherapy recommendations where appropriate with the addition of carboplatin [66–68].

It is strongly recommended to discuss and consider bilateral prophylactic mastectomy for certain mutations to prevent the risk of developing breast cancer. These include pathogenic variants in *BRCA1, BRCA2, PALB2, PTEN*, and *TP53* [7].

We strongly recommend consultation with a plastic surgeon and consideration of immediate simultaneous reconstruction for women who opt for prophylactic mastectomy for prevention. If the patient does not wish to pursue bilateral prophylactic mastectomy, then increased monitoring is recommended. NCCN guidelines recommend annual MRI and mammogram, alternated by six months, in additional to bi-annual physical examinations. Women should continue to perform monthly breast examinations [7].

### Surgical oncology

#### Breast

We strongly recommend clearly marking any specimen that is removed from the breast as well as clearly marking a mastectomy specimen as well. There are several methods by which to mark a specimen, including suture marking orientation versus using commercially available markers such as a margin map to clearly denote specimen orientation.

It is strongly recommended that a lumpectomy/partial mastectomy/tumorectomy is performed where possible if breast conservation is not contraindicated. Size of the tumor and size of the breast should be considered for an optimal cosmetic result in addition to optimal oncologic treatment. Multiple-randomized controlled trials with long-term follow-up have demonstrated no survival benefit to more aggressive surgery such as a mastectomy; therefore, breast conservation should be recommended where able [69]. Studies have also shown that women who are able to have breast conservation have higher satisfaction scores for cosmesis when compared to women who have undergone a mastectomy [70].

The use of pre-operative localization of tumors is a widely accepted technique utilized internationally for identification of non-palpable tumors. Lumpectomy is often not considered or possible, where pre-operative localization is not available. For this reason, we strongly recommend that pre-operative localization is available for surgeons to utilize breast conservation therapy.

Consider mastectomy in situations, where breast conservation is not feasible. This will also be addressed in the plastic and reconstructive surgery section; however, consider immediate reconstruction at the time of mastectomy if considered to be safe from an oncological standpoint.

Consider bilateral mastectomy in situations, where a genetic mutation is involved (see section 3.5). When bilateral mastectomy is performed for prophylactic reasons, consider immediate reconstruction if the patient so desires. Any immediate reconstruction is reasonable including implant-based reconstruction versus autologous tissue reconstruction.

#### Axilla (No clinical disease at time of presentation)

We strongly recommend a sentinel lymph biopsy in clinically node negative patients who are having upfront surgery. This involves patients with all clinical T status, with the exception of inflammatory disease and known node positive disease at time of clinical diagnosis.

It is strongly recommended that Tc99 and blue dye, either lymphazurin dye or methylene blue dye, are used together when performing an SLNB to decrease the false negative rate of sentinel lymph node identification. The use of two dyes has been shown to have a sentinel lymph node identification rate of 97% with a false negative rate of 9.8% [71–77]. In addition to this, ACOSOG Z0011 found that removal of three or more sentinel lymph nodes further reduced the false-negative rate in the setting of breast conservation [71].

We do not recommend an intraoperative frozen section at the time of an SLNB for clinically node negative (cN0) disease.

The need for further axillary surgery after the final pathology is available for the initial SLNB should be decided on the amount of axillary burden, clinical characteristics, and tumor characteristics [71, 78].

#### Axilla (treatment after neoadjuvant therapy)

For inflammatory breast cancer, we strongly recommend axillary dissection.

For cN0 patients pre-NAC, we strongly recommend a SLNB with the use of Tc99 and blue dye (either lymphazurin blue or diluted methylene blue dye) where technically possible.

For clinically and/or pathologically suspicious/positive lymph nodes (cN1/2) pre-NAC, we strongly recommend SLNB with Tc99 and blue dye if clinically down staged (by physical examination or imaging) with intraoperative frozen section and immediate axillary dissection only if persistently positive nodal disease is identified. We do not recommend immediate axillary dissection, rather an attempt at an SLNB per the ACOSOG 1071 data and ongoing Alliance 11202 trial [11, 79, 80].

We strongly recommend the removal of at least three lymph nodes during sentinel biopsy with Tc99 and lymphazurin blue dye or methylene blue dye (where available) to decrease the false negative rate [11, 79, 80].

We strongly recommend performing a CNB of all suspected lymph nodes pre-NAC. If possible, we recommend placing a clip/marker in the lymph node at the time of CNB for localization of this lymph node at the time of surgery, to ensure removal and decrease the false negative rate of the SLNB [11].

#### Registry

We strongly recommend the creation of a central database/registry, where data for each patient can be housed that will include diagnoses, histology, and pathology of the tumor, treatments, and outcomes including patient mortality. This will allow tracking of patient care and outcomes that will allow future progress in disease specific survival. Per NCCN guidelines, this is the mainstay of ensuring optimal patient care and outcomes.

#### Plastic and reconstructive surgery

We strongly recommend considering reconstruction in every woman (with few exceptions such as inflammatory breast cancer or rapidly growing tumors). Immediate reconstruction with a tissue expander should be considered in all women undergoing a mastectomy, when appropriate from an oncological standpoint. Consider pre-pectoral placement of tissue expanders when able [81–83].

We strongly recommend presentation of the patient case in a multidisciplinary fashion before surgery, particularly when post-mastectomy radiation therapy is planned or anticipated [83].

Consider autologous tissue reconstruction following mastectomy and post-mastectomy radiation therapy given concerns of possible complications of implant-based reconstruction in this setting such as infection or need for removal and subsequent delay of oncologic care [83].

A nipple or skin sparing approach to mastectomy should be considered when appropriate. Nipple sparing mastectomy should be considered when the tumor is >1 cm from the nipple [84].

We strongly recommend tracking patient operations and outcomes, clinical outcomes, as well as patient satisfaction outcomes [85–87].

### Medical oncology

#### Neoadjuvant chemotherapy for the early-stage breast cancer (Stage I/II)

We strongly recommend case presentation at a multidisciplinary tumor board before the start of surgical or systemic therapy.

We strongly recommend consideration of NAC for all palpable T2 and larger tumors that are triple-negative breast cancer (TNBC) or Her2 positive breast cancer. This allows for downstaging of breast and axillary disease and, further, can de-escalate the need for more aggressive surgical therapy. It also allows for physicians to use RCB class and PCR to tailor adjuvant therapies [88–90]. As such, we highly recommend consideration of NAC for downstaging of axillary disease N1 to attempt an SLNB and avoid an axillary dissection where possible [88, 89].

In addition, we highly recommend consideration of NAC to downstage large hormone positive or negative tumors to allow consideration of breast conservation. Randomized-controlled trials have shown that over 79% of patients had a clinical response with evidence of axillary nodal downstaging and increase in the rate of breast conservation [88, 89].

When starting NAC, we strongly recommend initiation within one month of diagnosis.

We strongly recommend considering dual Her2 therapy, when possible, for Her2 positive tumors [57, 91, 92].

We recommend staging scans with a bone scan and computerized tomography (CT) chest/abdomen/pelvis (C/A/P) before NAC.

We strongly recommend the completion of the diagnostic workup including placement of tumor clips/markers before starting chemotherapy. This will aid in the correct surgical management following chemotherapy.

We strongly recommend TDM1 in the adjuvant setting for women with Her2 positive cancers who do not achieve a PCR [93].

We strongly recommend Capecitabine in the adjuvant setting for women with TNBC who do not achieve a PCR [94].

We highly recommend consideration of clinical trials where appropriate.

#### Adjuvant considerations for the early-stage breast cancer (Stage I/II)

In the adjuvant setting, the surgical pathology report is primarily utilized to determine the need for chemotherapy. Often upfront surgery is used for TNBC or Her2 overexpressed tumors when they are screen detected or smaller and node negative (cT1N0). Consider less cytotoxic therapy in the adjuvant setting where appropriate for such tumors.

We strongly recommend the use of molecular profiling where appropriate and where financially feasible for estrogen positive tumors. This may include Oncotype Dx or MammaPrint [95–100].

We strongly recommend presentation of patient cases at a multidisciplinary tumor board before chemotherapy. Strong emphasis should be placed for fertility considerations in premenopausal women.

We strongly recommend consideration of Tamoxifen for premenopausal women with hormone positive disease or an aromatase inhibitor with ovarian suppression. Consideration of contraindications to use should be employed [101].

We recommend consideration of an aromatase inhibitor for postmenopausal women with hormone positive disease [7, 102].

#### Metastatic breast cancer

The goals of treating metastatic breast cancer are to extend survival and prevent disease progression while maintaining quality of life. The length of disease maintenance and extension of survival/life years depends on the stage of the disease, the number of affected organs, involvement of visceral organs versus bony disease, histologic characteristics of the tumor, and the general medical state of the patient. Metastatic breast cancer can present at the time of the primary disease or at time of recurrence, with or without a local component.

If it is recurrent disease, we strongly recommend biopsy of the recurrence to determine histologic markers and whether there is a change from the primary disease. This will help guide appropriate therapy. The organ for biopsy should be the easiest attainable target for biopsy with minimal discomfort to the patient, if possible.

We strongly recommend case presentation at a multidisciplinary tumor board before the start of surgical or systemic therapy.

Therapy is geared toward the histologic characteristics of the tumor, ER/PR, Her2 status, and patient factors, such as age, comorbidities, history of prior therapies, menopausal status, burden of disease, evidence of visceral crisis, and willingness to participate in further therapy. Therapeutic options for metastatic breast cancer include hormonal therapy, chemotherapy, anti-Her2 targeted therapy, targeted therapies, radiation therapy, surgery when applicable, and symptomatic/palliative therapies [103–109]. Surgery in this setting has not been shown to impact survival and should be used judiciously [103, 105, 109]. The treatment is individually determined, depending on the tumor characteristics, patient characteristics, and goals of care of the patient and their families.

#### Treatment of metastatic Her2-positive breast cancer

For patients who have documented Her2-positive metastatic breast cancer, intravenous (IV) Her2-targeted therapies, either alone or dual therapies with pertuzumab, should be considered if the patient has no significant comorbidities. A subcutaneous injectable format can be used where applicable and where able to be obtained; particularly for patients who cannot tolerate IV therapy [57].

We strongly recommend the consideration of clinical trials where available.

#### Treatment of metastatic ER-positive, Her2-negative breast cancer

The majority of breast disease is hormone positive and Her2-negative. Therefore, this is also a common subtype in metastatic breast cancer. The mainstay for the treatment of metastatic and hormone positive disease is hormone therapy. Chemotherapy can be considered if appropriate depending on disease burden and patient goals of life. Other therapies such as radiation should be considered for symptomatic disease.

#### Treatment of metastatic triple-negative breast cancer (TNBC)

The mainstay for treatment of metastatic TNBC is chemotherapy. In addition, bisphosphonates and Denosumab should be considered for bony metastatic disease, in addition to targeted therapy based on tumor characteristics. Clinical trial consideration is strongly recommended [90].

We strongly recommend the evaluation of PD-L1 expression to help guide therapy. Other therapies such as radiation should be considered for symptomatic disease.

#### Radiation oncology

We strongly recommend that all women with breast cancer who undergo breast conservation should consider adjuvant whole breast radiation therapy if under the age of 70. Strongly consider a boost to the lumpectomy cavity in the setting of breast conservation [69, 70, 110–112].

We recommend hypofractionation when possible and where feasible. Strongly consider 3D conformal therapy and intensity-modulated radiation therapy (IMRT) where appropriate [110, 112].

Following a mastectomy, consider radiation therapy for node positive disease, close or positive margins, high-risk disease, or medial tumors [113, 114].

We strongly recommend consideration of regional nodal radiation when appropriate.

Consider the omission of radiation therapy for women over the age of 70 with small, ER positive, clinically node negative tumors (cT1N0). The Cancer and Leukemia Group B (CALGB) study supports this [115].

Intraoperative radiation therapy does not have significant long-range studies currently to support widespread use. Use where appropriate and if technology is available [116].

To avoid a delay, timing of radiation therapy should not exceed 4–6 weeks following oncologic surgery [117].

### Young women with breast cancer

Young women with breast cancer are often defined as age less than or equal to 40 and comprise 5%–6% of the overall breast cancer population. This definition is consistent with previous guidelines [118, 119]. Although studies have shown that younger women can present with more aggressive tumors and have an increased risk of relapse, there is no clear indication that more aggressive therapy than indicated will affect outcome. International guidelines on the treatment of young women support this as well. We, therefore, recommend treating young women the same as older women with breast cancer when related to treatment [120].

### Breast cancer in pregnancy

Breast cancer in pregnancy is defined as a breast cancer diagnosis during pregnancy or in the first postpartum year. This is a rare occurrence and happens in approximately 1 in 3000 pregnant women and is the second most common malignancy affecting pregnancy.

We strongly recommend operative intervention where there is no need for NAC. The preferred timing for operative intervention is in the second trimester, to allow for completion of organogenesis in the first trimester.

We strongly recommend using Tc99 alone for the purposes of an SLNB when performing the procedure on a pregnant woman. Lymphazurin blue has been associated with a risk of allergic reactions and anaphylaxis, while methylene blue is associated with jejunal atresia during the first trimester and should, therefore, not be used in pregnancy [7, 121, 122].

We strongly recommend the use of NAC when appropriate based on tumor characteristics and patient factors. If administered, this should be initiated after the first trimester and should be completed by the 35th week of pregnancy. Chemotherapy administered during the first trimester or when organogenesis is taking place from the 4th–12th week of pregnancy, poses an elevated risk of fetal teratogenesis, and, for this reason, should be avoided [122–126].

Tamoxifen should be avoided in pregnancy as it is associated with a 20% risk of birth defects and is, therefore, contraindicated in pregnancy [122].

Surgical considerations should be driven by the timing of surgery. Due to the risk of fetal loss, this should be avoided in the first trimester when at all possible. When performed in the first and second trimesters, mastectomy is the general recommendation. To minimize the time under anesthesia, consideration should be given to delayed reconstructive surgery if possible [122–126].

Breast conservation remains an option for pregnant women if they can deliver the baby safely and then proceed to breast conservation. This should not cause a delay to radiation therapy and would therefore be a reasonable option. However, radiation therapy is not recommended during pregnancy.

We strongly recommend the involvement of high-risk maternal-fetal medicine specialists in addition to obstetricians in a multidisciplinary approach for optimal outcomes in pregnancy-related breast cancer.

While termination of pregnancy remains an option to the patient, a multidisciplinary approach to breast cancer care during pregnancy allows for care of both the baby and the mother.

## Discussion

The aim of this manuscript is to serve as a guideline for the care of breast cancer patients in BiH. This is the first multidisciplinary breast cancer consortium with the goal of establishing and publishing national guidelines. These recommendations are aimed to organize a standard of care that is expected for breast cancer patients, as established by well-defined international guidelines. Recommendations are evidence-based per multidisciplinary section and have considered regional and national resource availability. We hope that this will aid the health ministry in providing resources that might be absent. We are optimistic that the guidelines will encourage local providers to elevate the standard of care for breast cancer patients in BiH.

## Acknowledgments

We would like to acknowledge the assistance of Michelle L. Wallander in medical editing and reviewing. Funding support was provided by the Bosnian Education Fund from within the Levine Cancer Institute (Grant #5295).

**Conflicts of interest:** The authors declare no conflicts of interest.

**Funding:** Funding support was provided by the Bosnian Education Fund from within the Levine Cancer Institute (Grant #5295).
